# Commentary: Assessing Cognitive Abilities in High-Performing Cochlear Implant Users

**DOI:** 10.3389/fnins.2019.00564

**Published:** 2019-06-04

**Authors:** Ellen Andries, Vincent Van Rompaey, Paul Van de Heyning, Griet Mertens

**Affiliations:** ^1^Department of Otorhinolaryngology, Head and Neck Surgery, Antwerp University Hospital, Antwerp, Belgium; ^2^Experimental Lab of Translational Neurosciences and Dento-Otolaryngology, Faculty of Medicine and Health Sciences, University of Antwerp, Antwerp, Belgium

**Keywords:** cochlear implants, cognition, clinical outcome, working memory, RBANS-H

## Introduction

The interesting research of Hillyer et al. (2019) pointed out the burden of hearing loss and inadequate performance of cognitive tasks. Our research group focused on alleviating this burden (Claes et al., 2016, 2018a,b). Recent studies established an improvement of cognitive abilities after cochlear implantation in older adults (Jayakody et al., 2017; Claes et al., 2018a,b; Völter et al., 2018). The modality of presentation of cognitive test batteries is important when evaluating cognition in Cochlear Implant (CI) users. Their severe to profound hearing loss could possibly have a negative impact on the results of common, often auditory-only cognitive assessments (Zekveld et al., 2007; Dupuis et al., 2014; Jayakody et al., 2018; Hillyer et al., 2019). Hearing impairment can induce problems in hearing and understanding auditory presented instructions and stimuli. Therefore, there is a need for a cognitive test battery for CI recipients adjusted for hearing loss, for example by means of visual support.

## Cognitive Assessment of Hearing Impaired Listeners

Hillyer et al. (2019) evaluated cognition in CI recipients with high speech perception scores in quiet (scores at or above 60% on AzBio Sentences Test) using visual vs. auditory-visual (auditory input and lipreading) presentation. Six tests of the Woodcock-Johnson-IV WJ-IV test battery were administered. Two of those tests were used to assess the impact of presentation modality. In the visual working memory task, subjects had to repeat a set of numbers that were presented to them visually on a card for one second per number, backwards. The auditory-visual working memory task was similar but auditory input and/or lipreading abilities had to be used, as numbers were read aloud instead of presented on a card. Hillyer et al. (2019) hypothesized that working memory could be associated with speech perception in adult CI users. They assumed that visual item presentation could provide a more accurate measure of CI recipients' cognition because it accounts for audibility. Hence, a visually presented cognitive test battery could be more appropriate to examine the connection between speech perception and cognition in CI users. The results of Hillyer et al. (2019) confirm that cognitive assessment in high-performing CI recipients could be improved through the application of visual presentation.

Recent studies of our research group also acknowledge the importance of visual support during cognitive evaluation of CI users (Claes et al., 2016, 2018a,b). They could provide a valuable complement to the article of Hillyer et al. (2019). Claes et al. (2016, 2018a,b) adjusted the Repeatable Battery for the Assessment of Neuropsychological Status (RBANS) to a version that allowed testing hearing impaired subjects (RBANS-H) (Randolph et al., 1998). They used a PowerPoint presentation to visually support oral instructions during assessment and to enable an exact timing of stimulus presentations. RBANS-H measures are similar to the Woodcock-Johnson-IV WJ-IV measures used by Hillyer et al. (2019). Both test batteries measure working memory/immediate memory, attention and spatial relations/visuospatial. RBANS-H could be an addition to Woodcock-Johnson-IV WJ-IV as the test battery also comprises delayed memory and language measures. Moreover, RBANS-H is suited and was already used to assess CI recipients with low speech perception scores (Claes et al., 2016, 2018a,b).

The Hearing Impaired Montreal Cognitive Assessment (HI-MoCA), the Cambridge Neuropsychological Test Automated Battery (CANTAB) and the multimodular computer-based cognitive test battery developed at the Institute for Work, Learning and Aging (ALA) in Dortmund, Germany (ALAcog) also use visual presentation to assess cognition in hearing impaired subjects (Zekveld et al., 2007; Lin et al., 2017; Jayakody et al., 2018; Völter et al., 2018). Thus, those tests could supplement Hillyer et al. (2019) as well. HI-MoCA can be used as a cognitive screening tool for subjects with hearing loss, whereas ALAcog and CANTAB are cognitive test batteries covering multiple cognitive domains similar to RBANS-H. HI-MoCA uses a PowerPoint presentation to provide visual instructions, ALAcog and CANTAB are computerized test batteries. These tests are all administered without any verbal cues and can be used in CI recipients with poor speech perception scores.

Additionally, objective electrophysiological assessments such as event-related brain potentials (ERP) could also complement the methods used by Hillyer et al. (2019). Studies demonstrated that the peak latency of the cognitive potential (P300) is prolonged in subjects with cognitive impairment compared to normal aging subjects (Squires et al., 1980; Vecchio and Määttä, 2011). P300 can be elicited by both visually and auditory presented stimuli, so it is suitable for hearing impaired patients (Polich, 2004). Hence, it could be valuable in the assessment of changes in cognitive functioning of CI recipients. Several studies have already used the component in the objective assessment of auditory cognition and speech perception in CI users (Groenen et al., 2001; Nager et al., 2007; Henkin et al., 2014; Finke et al., 2016).

## Conclusion

RBANS-H, HI-MoCA, CANTAB and ALAcog are suitable for the assessment of cognitive abilities in hearing impaired subjects because of using visual support during administration. P300 measurement could be a useful objective method to assess changes of cognitive abilities in CI recipients. Consequently, those tests could provide a valuable complement to the research of Hillyer et al. (2019), highlighting the importance of visual presentation in the evaluation of cognition in CI recipients.

## Author Contributions

EA drafted the manuscript. GM, PV, and VV critically revised the manuscript. All authors read and approved the final manuscript.

### Conflict of Interest Statement

The authors declare that the research was conducted in the absence of any commercial or financial relationships that could be construed as a potential conflict of interest.

## References

[B1] Claes A. J. Mertens G. Gilles A. Hofkens-Van den Brandt A. Fransen E. Van Rompaey V.. (2016). The repeatable battery for the assessment of neuropsychological status for hearing impaired individuals (RBANS-H) before and after cochlear implantation: a protocol for a prospective, longitudinal cohort study. Front. Neurosci. 10:512. 10.3389/fnins.2016.00512 27895549 PMC5108794

[B2] Claes A. J. Van de Heyning P. Gilles A. Hofkens-Van den Brandt A. Van Rompaey V. Mertens G. (2018b). Impaired cognitive functioning in cochlear implant recipients over the Age of 55 Years: A cross-sectional study using the repeatable battery for the assessment of neuropsychological status for hearing-impaired individuals (RBANS-H). Front. Neurosci. 12:580. 10.3389/fnins.2018.00580 30197584 PMC6117382

[B3] Claes A. J. Van de Heyning P. Gilles A. Van Rompaey V. Mertens G. (2018a). Cognitive performance of severely hearing-impaired older adults before and after cochlear implantation: preliminary results of a prospective, longitudinal cohort study using the RBANS-H. Otol. Neurotol. 39, e765–e73. 10.1097/MAO.0000000000001936 30153132

[B4] Dupuis K. Pichora-Fuller M. K. Chasteen A. L. Marchuk V. Singh G. Smith S. L. (2014). Effects of hearing and vision impairments on the montreal cognitive assessment. Aging Neuropsychol. Cogn. 22, 1–25. 10.1080/13825585.2014.968084 25325767

[B5] Finke M. Büchner A. Ruigendijk E. Meyer M. Sandmann P. (2016). On the relationship between auditory cognition and speech intelligibility in cochlear implant users: an ERP study. Neuropsychologia 87, 169–181. 10.1016/j.neuropsychologia.2016.05.019 27212057

[B6] Groenen P. A. P. Beynon A. J. Snik A. F. M. Broek P. V. D. (2001). Speech-evoked cortical potentials recognition in cochlear implant users and speech. Scand. Audiol. 30, 31–40. 10.1080/010503901750069554 11330917

[B7] Henkin Y. Yaar-Soffer Y. Steinberg M. Muchnik C. (2014). Neural correlates of auditory-cognitive processing in older adult cochlear implant recipients. Audiol. Neurootol. 19(Suppl. 1), 21–26. 10.1159/000371602 25733362

[B8] Hillyer J. Elkins E. Hazlewood C. Watson S. D. Arenberg J. G. Parbery-Clark A. (2019). Assessing cognitive abilities in high-performing cochlear implant users. Front. Neurosci. 12:1056. 10.3389/fnins.2018.01056 30713488 PMC6346679

[B9] Jayakody D. M. P. Friedland P. L. Eikelboom R. H. Martins R. N. Sohrabi H. R. (2018). A novel study on association between untreated hearing loss and cognitive functions of older adults: baseline non-verbal cognitive assessment results. Clin. Otolaryngol. 43, 182–191. 10.1111/coa.12937 28710824

[B10] Jayakody P. D. M. Friedland L. P. Nel N. E. Martins D. R. Atlas R. M. Sohrabi R. H. (2017). Impact of cochlear implantation on cognitive functions of older adults: pilot test results. Otol. Neurotol. 38, e289–e95. 10.1097/MAO.0000000000001502 28806341

[B11] Lin V. Y. W. Chung J. Callahan B. L. Smith L. Gritters N. Chen J. M.. (2017). Development of cognitive screening test for the severely hearing impaired: hearing-impaired MoCA. Laryngoscope 127, S4–S11. 10.1002/lary.26590 28409842

[B12] Nager W. Munte T. F. Bohrer I. Lenarz T. Dengler R. Mobes J.. (2007). Automatic and attentive processing of sounds in cochlear implant patients - electrophysiological evidence. Restor. Neurol. Neurosci. 25, 391–396. 17943014

[B13] Polich J. (2004). Clinical application of the P300 event-related brain potential. Phys. Med. Rehab. Clin. North Am. 15, 133–161. 10.1016/S1047-9651(03)00109-8 15029903

[B14] Randolph C. Tierney M. C. Mohr E. Chase T. N. (1998). The repeatable battery for the assessment of neuropsychological status (RBANS): preliminary clinical validity. J. Clin. Exp. Neuropsychol. 20, 310–319. 10.1076/jcen.20.3.310.823 9845158

[B15] Squires K. C. Chippendale T. Wrege K. Goodin D. S. Start A. (1980). “Electrophysiological assessment of mental function in aging and dementia,” in Determining the Effects of Aging on the Central Nervous System Free University of Berlin, ed Gurski G. E. (Berlin: Schering HE), 93–104. 10.1037/10050-009

[B16] Vecchio F. Määttä S. (2011). The use of auditory event-related potentials in Alzheimer's disease diagnosis. Int. J. Alzheimers Dis. 2011:653173. 10.4061/2011/653173 21629759 PMC3100636

[B17] Völter C. Götze L. Dazert S. Falkenstein M. Thomas J. P. (2018). Can cochlear implantation improve neurocognition in the aging population? Clin. Interv. Aging 13:701–712. 10.2147/CIA.S160517 29719382 PMC5916259

[B18] Zekveld A. A. Deijen J. B. Goverts S. T. Kramer S. E. (2007). The relationship between nonverbal cognitive functions and hearing loss. J. Speech Lang. Hear. Res. 50, 74–82. 10.1044/1092-4388(2007/006) 17344549

